# Pomegranate Supplementation Protects against Memory Dysfunction after Heart Surgery: A Pilot Study

**DOI:** 10.1155/2013/932401

**Published:** 2013-09-16

**Authors:** Susan A. Ropacki, Sapna M. Patel, Richard E. Hartman

**Affiliations:** ^1^Veterans Affairs Palo Alto Health Care System, PTRP-MB2A, 3801 Miranda Aveune, Palo Alto, CA 94304, USA; ^2^Department of Psychology, Loma Linda University, 11130 Anderson Street, Loma Linda, CA 92592, USA

## Abstract

Memory dysfunction is a common complaint following heart surgery and may be related to a diffuse ischemic state induced by microemboli dislodged during the procedure. Ischemia can induce damage by a number of mechanisms, including oxidative stress. Because pomegranates contain a variety of polyphenols with antioxidant and other potentially beneficial effects, we tested whether supplementation with a pomegranate extract before and after heart surgery could protect against postoperative cognitive dysfunction. Patients undergoing elective coronary artery bypass graft and/or valve surgery were given either 2 g of pomegranate extract (in 2 POMx pills) or placebo (pills containing no pomegranate ingredients) per day from one week before surgery to 6 weeks after surgery. The patients were also administered a battery of neuropsychological tests to assess memory function at 1 week before surgery (baseline), 2 weeks after surgery, and 6 weeks after surgery. The placebo group had significant deficits in postsurgery memory retention, and the pomegranate treatment not only protected against this effect, but also actually improved memory retention performance for up to 6 weeks after surgery as compared to presurgery baseline performance.

## 1. Introduction

Postoperative cognitive dysfunction (POCD) has been observed in patients following surgery with general anesthesia [1], especially following heart surgery [2]. The well-known case study of a patient who developed relatively severe and permanent amnesia following open-heart surgery, presumably due to ischemic hippocampal damage suffered during the surgery, is a striking illustration of these deficits [3]. Coronary artery bypass grafting (CABG) and valve replacement surgery have been shown by a number of studies to induce prolonged POCD [4–6]. Proposed mechanisms for POCD following heart surgery include general hypoperfusion of the brain (global ischemia), leading to critically low levels of oxygen and glucose throughout the brain, and focal ischemia due to microembolic particles released into the vasculature during surgery [7]. Additionally, inhaled anesthetics have been shown to induce the formation of amyloid-beta, a potentially neurotoxic protein linked to Alzheimer's disease and acute cognitive deficits, in the brain [8–10].

Increasing evidence suggests that dietary manipulations may reduce neuropathology and/or cognitive deficits in a number of disease states. In particular, foods with high concentrations of bioactive polyphenols have been shown to be neuroprotective against rodent models of stroke [11] and Alzheimer's disease [12, 13]. These neuroprotective polyphenols include epigallocatechin-3-gallate (green tea), curcumin (turmeric), resveratrol (red wine, grape juice), quercetin (grapefruit), and ellagic acid (raspberries, pomegranates). For example, dietary supplementation of pregnant mice with pomegranate juice was shown to protect against brain damage in the hippocampus when the neonatal mice were subjected to hypoxic-ischemic brain injury (a model of insufficient blood supply to the brain during birth) [11]. In other studies with transgenic mouse models of Alzheimer's disease, pomegranate juice reduced both neuropathology and behavioral deficits [12, 13]. The potential protective mechanisms of polyphenols include antioxidant properties and suppression of inflammatory and other pathways [14], as well as inhibiting accumulation of amyloid-beta in the brain [12]. Polyphenolic substances may also be transformed into potentially beneficial metabolic by-products by colonic microflora (microbes in the gut) [15]. It should be noted that whole foods often contain several phenolic substances, and research suggests that synergistic activity between multiple polyphenols may prove more beneficial than treatment with isolated polyphenols [16].

Pomegranate juice contains relatively high concentrations of polyphenolic substances [17]. Human studies have detected these substances and their metabolic by-products in the blood after oral administration [18–21], and data from rodent studies suggests that they can positively affect the brain [11–13, 22, 23]. Because of its neuroprotective and cardiovascular-enhancing properties, and because it is relatively inexpensive (compared to pharmaceuticals) and well tolerated, pre- and postsurgical dietary supplementation with pomegranate may prove to be effective in preventing or reducing post-operative cognitive deficits.

Cardiac surgery patients are the ideal population to test this hypothesis because of their relatively high risk of POCD. It has been shown by several animal studies that both global and focal ischemia can induce brain damage and learning/memory deficits (e.g., see [24]). Therefore, reduction of the brain damage and/or dysfunction putatively caused by cardiac surgery may prevent the associated cognitive deficits. 

Although findings on cognitive declines have been consistent in CABG and heart valve surgery research, preventive or treatment options for cardiac surgery-related cognitive dysfunction have not been examined extensively. In recent years, there has been a great deal of emphasis on the beneficial effects of pomegranate on health, including diseases of the brain and the heart. Few studies, however, have explored the impact of pomegranate on cognitive functions. Thus, the current study is unique in its exploration of the effects of pomegranate on various health parameters and its effectiveness in reducing cognitive declines (specifically memory) after heart surgery. 

Heart disease is the leading cause of death in the US and is associated with cognitive declines. CABG and heart valve surgery have been employed as treatments to help restore cardiac functioning to an optimal level and have decreased mortality rates associated with heart disease. However, there is evidence of relatively long-term memory deficits in the postoperative period [25]. Nutritional supplements can successfully moderate some of these heart disease risk factors, so we speculated that they might also ameliorate the negative cognitive consequences of heart disease and surgical interventions as well. Preventive options for the cognitive declines observed after cardiac surgery have not been examined extensively. The aim of the current study was to examine the impact of pomegranate supplementation on memory performance after cardiac surgery. This was examined by comparing the results of baseline neuropsychological tests for immediate memory, delayed memory, and retention in treatment and placebo groups to post-operative test results.

## 2. Materials and Methods

### 2.1. Subjects

The subjects were elective cardiac surgery (CABG and/or valve repair/replacement) patients who were recruited from the Loma Linda University International Heart Institute at the time they presented for a preoperative evaluation and scheduled surgery. Exclusion criteria included less than 6 years of education, a history of previous cardiac surgery, planned concomitant noncoronary procedures, history of allergy to pomegranates, history of head injury, neurodegenerative disease or neurologic condition with known cognitive impact (e.g., Alzheimer's disease, muscular sclerosis), history of drug or alcohol abuse, DSM-IV psychiatric disorder, active renal disease (serum creatinine concentration >2.0 mg per deciliter), active liver disease, or left ventricular ejection fraction of less than 20%.

### 2.2. Treatment

Subjects were randomly assigned to the pomegranate or placebo groups. Participants in the pomegranate group (*n* = 5) were given two POMx (Pom Wonderful, CA, USA) capsules (one in the morning and one in the evening). Each 1 g capsule contained a concentrated blend of the polyphenols derived from 240 mL of pomegranate juice (~375 mg punicalagins, 93 mg anthocyanins, 29 mg ellagic acid, and 100 mg other tannins). We chose a dose of two pills per day because doses of 1-2 have proven safe and effective in other human studies [20, 26–28]. Participants in the placebo group (*n* = 5) were given two placebo capsules that looked identical to the PomX pills but contained no pomegranate ingredients. 

### 2.3. Data Collection

Information was gathered on a number of biomedical risk factors and demographic variables, including prior history of smoking, hypertension, cholesterol, diabetes, education, and social support. An IQ estimate was calculated using the sum of scaled scores from the Wechsler Adult Intelligence Scale–III (WAIS-III; Wechsler, 1997) Vocabulary and Matrix Reasoning subtests (ERSET operations manual).

The mean age of the pomegranate group was approximately 62.4 years, the mean number of years of education was 15.3 years, and the mean estimated IQ was 100.6. The mean age of the placebo group was approximately 70.2 years, the mean number of years of education was 15 years, and the mean IQ estimate was 106.6. CABG and valve replacement surgeries were equally represented in each group (see Table 1). Treatment began one week before surgery and continued through 6 weeks after surgery. Trained graduate students in psychology, or the licensed clinical psychologist associated with this study, performed all cognitive assessments under standardized conditions. All examiners and subjects were blinded to treatment group. 

### 2.4. Neuropsychological Tests

Tests to assess memory function were administered just prior to the first pomegranate pill administration one week before surgery (baseline), 2 weeks after surgery (short term), and 6 weeks after surgery (long term). The memory tests included the WAIS-III Digit Span subtest, Hopkins Verbal Learning Test-Revised (HVLT-R), the Rey Complex Figure Test with recognition trial (RCFT), the Logical Memory subtest of Wechsler Memory Scales (WMS-III), and the Brief Visuospatial Memory Test-Revised (BVMT-R).

The WAIS-III Digit Span subtest was administered as a measure of working memory. It is comprised of a forward digit span repetition task, in which the respondent repeats back an increasingly long series of single-digit numbers and a backward digit span repetition task, in which the respondent repeats an increasingly long series of single-digit numbers in reverse order. A total score is calculated based on the number of correctly repeated number strings. 

The HVLT-R assesses verbal learning and memory. It is a list-learning task that consists of three immediate recall trials, a 20-minute delayed recall trial and a recognition trial, consisting of 12 words. There is an immediate recall score (total of all three trials), learning score (highest of T2 or T3 minus T1), delayed memory score which is the total number of words recalled after the delay, and a recognition score which is the total number of words recalled in a list of target words and distractors. A retention score representing the percentage of information retained was created using the following formula: {[(learning score − delayed recall score)/learning score] × 100} − 100. 

The RCFT assesses visuospatial memory. It consists of a copy trial, an immediate recall trial (3 minutes), a delayed recall trial (30 minutes), and a recognition trial. Scores include a copy score (which reflects the accuracy of the original figure and the time required to copy the figure), immediate and delayed recall scores (assess the amount of information retained over time), and the number of items correctly and incorrectly identified on a recognition task. A retention score representing the percentage of information retained was calculated using the following formula: {[(learning score − delayed recall score)/learning score] × 100} − 100. 

The WMS-III Logical Memory subtest assesses contextual verbal learning and memory. It consists of two stories that are read out loud by the examiner. The first story is read once and the second story is read twice. Participants are to recall the stories immediately and after a 25–35-minute delay and are given a yes/no recognition trial following the delays. Scores include an immediate recall and delayed recall (reflecting the amount of information retained over time) for all the details of both stories, learning score for the second story, and a recognition score. A retention score representing the percentage of information retained was created using the following formula: {[(learning score − delayed recall score)/learning score] × 100} − 100. 

The BVMT-R assesses visuospatial memory. It consists of three trials in which a display of 6 figures is presented for 10 seconds and participants are required to draw the figures in their correct locations on the page after each trial. They are required to recall these figures after a 20-minute delay and are given a yes/no recognition trial following the delays. Scores include immediate recall (total of all trials), delayed recall, learning, and recognition. A retention score representing the percentage of information retained was created using the following formula: {[(learning score − delayed recall score)/learning score] × 100} − 100. 

Raw scores on the selected memory test variables were converted into *z*-scores using normative data (taking into account age and educational level), and average composite scores were calculated for immediate memory, delayed memory, and retention by averaging the *z*-scores from each test for each of these domains. Each patient's short-term (2 weeks after surgery) and long-term (6 weeks after surgery) scores were then normalized to their baseline (1 week before surgery) scores to assess whether scores improved or deteriorated following surgery. Statistical analyses were carried out using the SPSS (PASW Statistics 18) statistical software package (SPSS, Inc., Chicago, IL, USA). A repeated-measures between-groups analysis of variance (ANOVA) was conducted using short- versus long-term working memory data and composite scores for immediate memory, delayed memory, and retention to examine differences between and within groups at each post-operative time point.

## 3. Results and Discussion

### 3.1. Neuropsychological Tests

**Figure 1:**
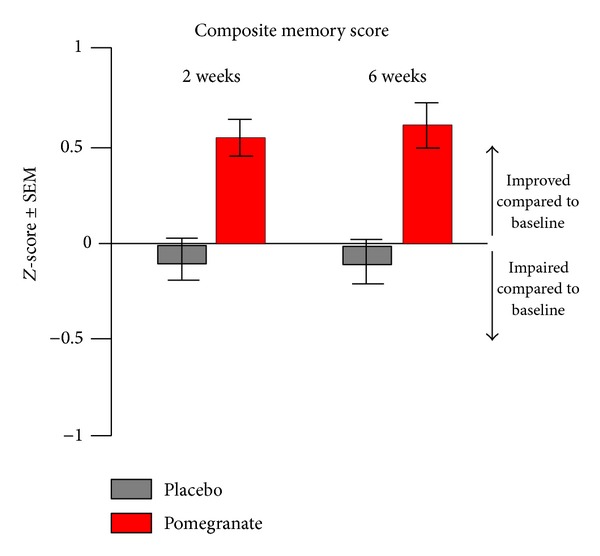
Pooling data across all four memory domains revealed a trend for an improvement from presurgery baseline scores for the pomegranate group at both short- and long-term time points, whereas the placebo group performed slightly worse than baseline at both time points.

**Figure 2:**
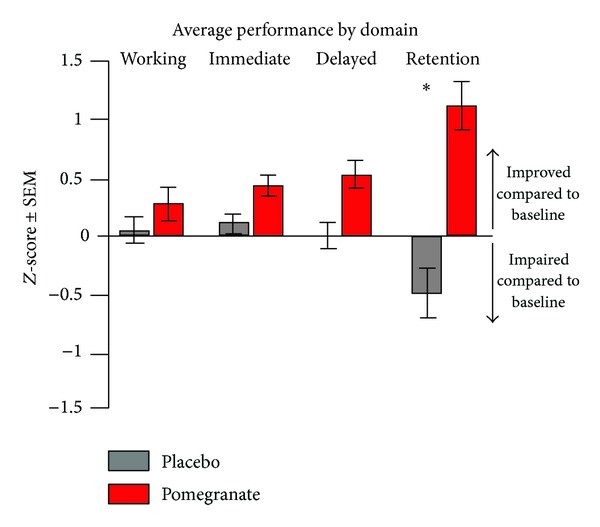
Pooling data across both time points revealed a specific retention memory deficit in the placebo group after surgery, whereas the pomegranate group's performance across all domains improved after surgery (*p < .05).

**Figure 3:**
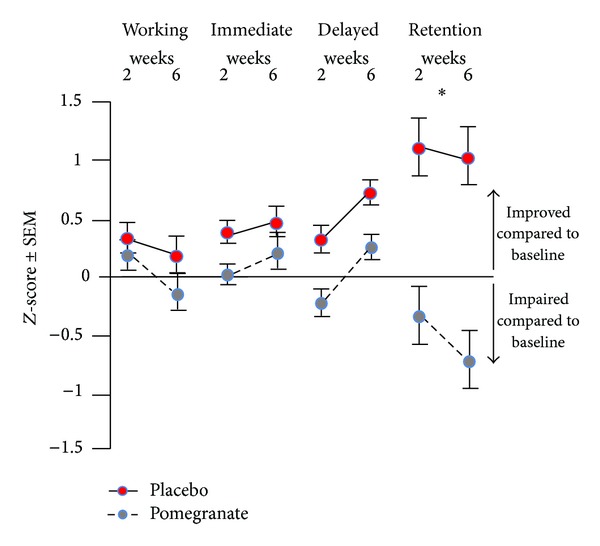
Working memory, immediate memory, and delayed memory all improved at least slightly from baseline at both short- and long-term time points for the pomegranate group. Scores for the placebo group on these domains did not change significantly from baseline. Retention scores for the placebo group were impaired compared to baseline 2 weeks after surgery and continued to decline for up to 6 weeks after surgery. Retention scores for the pomegranate group were significantly improved from baseline at both 2 and 6 weeks after surgery (*p* < .05).

There were no significant differences between groups on demographic factors such as age, education, estimated IQ, or any of the surgical or perioperative variables available on participants (see Table 1). Pooling data across all four memory domains (working, immediate, delayed, and retention) revealed a trend for an improvement from presurgery baseline scores for the pomegranate group at both short- and long-term time points, whereas the placebo group was slightly impaired compared to baseline at both time points (Figure 1). Additionally, pooling data across both time points revealed a significant and striking difference, in that *retention* was the memory domain most severely impacted by the surgery. The pomegranate treatment not only protected against this effect, but also seemed to improve retention performance from presurgery baseline performance (*F*[3, 24] = 3.03, *p* < .05; Figure 2). When domain and time point were analyzed together, it became clear that digit span (working memory), immediate memory, and delayed memory all improved at least slightly from baseline at both short- and long-term time points for the pomegranate group. In contrast, scores for the placebo group on these domains did not change significantly from baseline (Figure 3). However, retention scores for the placebo group were worse than baseline 2 weeks after surgery and continued to decline for up to 6 weeks after surgery. In contrast, retention scores for the pomegranate group were significantly improved from baseline at both 2 and 6 weeks after surgery (*F*[1, 8] = 5.64, *p* < .05; Figure 3).

These results suggest that the post-operative memory dysfunction following heart surgery consists mostly of retention issues rather than problems with working memory, immediate memory, or delayed memory. Retention scores on these neuropsychological tests reflect how much information is recalled relative to how much was actually learned and thus provides a measure of memory consolidation. In contrast, working memory (digit span) scores reflect the ability to attend and to maintain information for use in a task, immediate memory scores reflect how much information is learned in the short term, and delayed memory scores reflect how much information is recalled relative to how much information was actually presented.

### 3.2. Potential Mechanisms

**Figure 4:**
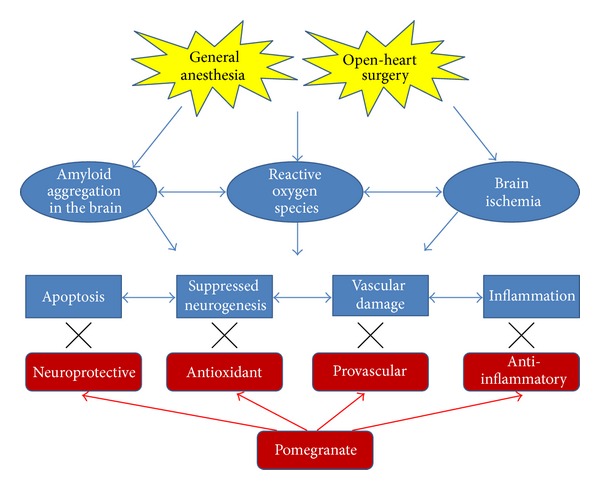
Pomegranates may protect the brain (and therefore memory) from the effects of open-heart surgery by a number of potential mechanisms. General anesthesia can induce amyloid aggregation in the brain and the surgery can induce brain ischemia, both leading to reactive oxygen species (e.g., free radicals). These insults may cause neuronal cell death (apoptosis), suppression of the birth of new neurons (neurogenesis), vascular damage, and inflammation in the brain. Pomegranate's antiapoptotic, antioxidant, anti-inflammatory, and provascular (via nitric oxide synthase) properties may protect against those effects.

The data also suggest that supplementation with pomegranate polyphenols can protect against surgery-induced retention deficits and may actually *improve* memory retention even after heart surgery. The specific mechanisms by which pomegranate polyphenols affect memory are currently unknown. In addition to the neuroprotective properties described above, pomegranates have been shown to improve a number of cardiovascular measures, including lipid metabolism and blood pressure. For example, pomegranate juice reduced low-density lipoprotein (LDL) aggregation, oxidative stress [14], serum angiotensin converting enzyme, and systolic blood pressure in hypertensive patients [29]. In patients with carotid artery stenosis, pomegranate juice reduced LDL oxidation, atherosclerosis, and systolic blood pressure [30]. In patients with ischemic coronary heart disease, pomegranate juice (PJ) decreased stress-induced ischemia but had no effect on blood sugar levels or blood pressure [31]. Studies with diabetic patients have shown that PJ decreased blood lipid oxidation, total cholesterol, and LDL cholesterol with no effects on high-density lipoprotein (HDL) or serum triacylglycerol [32]. A study with healthy volunteers showed that PJ decreased blood platelet aggregation/clotting [33]. However, a study with chronic obstructive pulmonary disease patients found no effects on respiratory function or lipid profile. In animal studies, PJ was shown to reduce LDL aggregation [14] and atherosclerosis [34] in hypercholesterolemic mice and improved cardiac lipid metabolism in a rat model of diabetes. It is possible that some of the vascular effects of PJ are due to its effects on nitric oxide synthase in the vasculature [34]. Also, PJ has been shown to inhibit cytochrome P450 3A (CYP3A), an enzyme involved in the regulation of blood vessel tone. However, unlike grapefruit juice, pomegranate juice has not been shown to inhibit drug clearance in humans by suppression of CYP3A [35] or CYP2C9 [36]. Moreover, multiple studies have shown that PJ also has anticancer [27, 37] and antimicrobial properties [38]. See Figure 4 for an illustration of potential mechanisms for the protective actions of pomegranate after open heart surgery.

### 3.3. Implications

The most important implication for patients is that if we are able to offset the amount of memory decline immediately after surgery, then their long-term prognosis may be better. Alternatively, there are important implications for both patients and healthcare personnel. Several factors can prolong intensive care and length of hospital stay, including intraoperative cerebral oxygen desaturation. If pomegranate is indeed targeting hypoxic mechanisms, then it may lead to shorter intensive care unit and hospital stays, thereby decreasing hospital costs and costs to the healthcare system. Another general implication for individuals is to increase engagement in health behaviors that are easily incorporated into daily life, are relatively free of negative side effects, and are comparatively inexpensive, to limit the number of factors (e.g., hypertension, high cholesterol, and smoking) that put them at greater risk for heart disease necessitating cardiac surgery and the subsequent cognitive deficits. Ultimately, continued research would lead to better treatments that improve the outcome of cardiac surgery and the cognitive well-being of the multitude of men and women who undergo these types of surgeries.

## 4. Conclusions

 Other studies using pomegranate have focused on its pro-cardiovascular [14, 29–35], anti-cancer [27, 37], and antimicrobial [38] properties. In 2006, we were the first to show that pomegranate can improve cognitive functions in mice [12]. In this study, we are the first to show that pomegranate can improve cognitive functions in humans. We have generated intriguing data suggesting that pomegranate polyphenols may provide long-lasting protection from heart surgery-induced memory retention deficits. We showed a clear pattern of postsurgical memory improvement in the pomegranate group across all of the memory domains that we tested, with an especially strong and long-lasting protective effect in the memory retention domain. It remains possible that the extra statistical power provided by a larger sample size may allow for the detection of statistically significant improvements across the remaining memory domains as well. It is also possible that extending the pre- and/or postsurgical treatment regimen may provide added benefit.

### 4.1. Future Directions

 The results of this study also raise a number of other interesting questions. Although we did not analyze neuropsychological data from other cognitive domains, it is possible that one or more other mental faculties could be negatively impacted by heart surgery and/or positively impacted by pomegranate treatment. Furthermore, we initiated treatment before the heart surgery. It remains to be seen whether starting treatment after surgery would have strong (if any) effects and also whether the dose we chose was the most effective dose. Would a larger dose provide more benefits and/or would a smaller dose prove equally as effective? Also, pomegranates were chosen because of their high polyphenolic content. Would other polyphenol-rich fruits and vegetables have a similar effect? Will isolated polyphenolic compounds provide as many beneficial effects as the combined range of compounds found in the whole fruit? Finally, future studies should analyze additional information regarding length of hospital stay, possible complications, preoperative and postoperative use of medications like beta-blockers and statins, and evaluation for delirium. Future clinical studies may provide the answers to these questions, and animal or *in vitro* studies may help to elucidate the molecular mechanisms behind the neuroprotective properties of polyphenols. Finally, perhaps the most intriguing prospect raised by this study is the possibility that dietary supplementation with pomegranate polyphenols can *improve* cognition. If this treatment can improve memory retention performance for individuals who have undergone a surgical treatment shown to cause a decline in performance, perhaps this treatment can improve the cognitive function of healthy individuals.

## Figures and Tables

**Table 1:** Demographic and perioperative data for the overall study sample by treatment group.

	All	Pomegranate	Placebo	Signif.
Age in years (mean ± standard deviation)	66.3 ± 8.9	62.4 ± 8.6	70.2 ± 8.1	*p* = .178
Years of education (mean ± standard deviation)	15.1 ± 1.96	15.3 ± 2.2	15.0 ± 2.0	*p* = .864
IQ estimate (mean ± standard deviation)	103.6 ± 17.0	100.6 ± 20.5	106.6 ± 14.4	*p* = .607
Gender (%)				*χ* ^2^ = .444
Male	80	100	60	
Female	20	0	40	
Ethnicity (%)				*χ* ^2^ = 1.00
Caucasian	60	60	60	
Hispanic	20	20	20	
Asian	20	20	20	
Marital status (%)				*χ* ^2^ = .444
Married	88.9	75	100	
Single	0	0	0	
Divorced	11.1	25	0	
Widowed	0	0	0	
Diabetes (%)				*χ* ^2^ = .524
Yes	55.6	60	25	
No	44.4	40	75	
High cholesterol (%)				*χ* ^2^ = .444
Yes	88.9	100	75	
No	11.1	0	25	
Hypertension (%)				*χ* ^2^ = .167
Yes	77.8	100	50	
No	22.2	0	50	
Smoking history (%)				*χ* ^2^ = .048
Yes	44.4	80	0	
No	55.6	20	100	
Surgery type (%)				*χ* ^2^ = .444
CABG	60	60	60	
Valve	30	40	20	
CABG + valve	10	0	20	
CPB minutes (mean ± standard deviation)	142.4 ± 48.1	151.3 ± 50.6	107.0 ± 0.0	*χ* ^2^ = .491
Cross-clamp minutes (mean ± standard deviation)	104.5 ± 50.7	127.5 ± 44.4	58.5 ± 4.7	*χ* ^2^ = .120
